# A Method of Spatial Mapping and Reclassification for High-Spatial-Resolution Remote Sensing Image Classification

**DOI:** 10.1155/2013/192982

**Published:** 2013-12-16

**Authors:** Guizhou Wang, Jianbo Liu, Guojin He

**Affiliations:** ^1^Institute of Remote Sensing and Digital Earth, Chinese Academy of Sciences, Beijing 100094, China; ^2^University of Chinese Academy of Sciences, Beijing 100049, China

## Abstract

This paper presents a new classification method for high-spatial-resolution remote sensing images based on a strategic mechanism of spatial mapping and reclassification. The proposed method includes four steps. First, the multispectral image is classified by a traditional pixel-based classification method (support vector machine). Second, the panchromatic image is subdivided by watershed segmentation. Third, the pixel-based multispectral image classification result is mapped to the panchromatic segmentation result based on a spatial mapping mechanism and the area dominant principle. During the mapping process, an area proportion threshold is set, and the regional property is defined as unclassified if the maximum area proportion does not surpass the threshold. Finally, unclassified regions are reclassified based on spectral information using the minimum distance to mean algorithm. Experimental results show that the classification method for high-spatial-resolution remote sensing images based on the spatial mapping mechanism and reclassification strategy can make use of both panchromatic and multispectral information, integrate the pixel- and object-based classification methods, and improve classification accuracy.

## 1. Introduction

With the development of improved sensors and powerful computation technology, high-spatial-resolution remote sensing data have been more easily acquired and widely applied [1]. High-spatial-resolution remote sensing images contain more information and have increased the detail at which earth observations can be made. The abundance of information, on one hand, has promoted the application of remote sensing methods but, on the other, brings new technological challenges to the data analysis. One challenge is that traditional image classification technology can no longer satisfy the needs of high-spatial-resolution remote sensing image classification.

High-spatial-resolution remote sensing imagery, such as SPOT-5, IKONOS, and QuickBird, has been used in many fields in recent years [2]. They have been applied for urban planning, urban change detection, tree canopy mapping, ecological environment monitor, precision agriculture, and so forth [3]. The main difference between a high-spatial-resolution remote sensing image and a low- or medium-resolution remote sensing image is that the high-spatial-resolution image provides more useful information, such as shape and texture. Therefore, the extraction of geographical information from a high-spatial-resolution satellite image is topical [2].

The traditional method of classification for high-spatial-resolution images has been proven to have several drawbacks, such as low classification accuracy, the derivation of very limited spatial information, and salt and pepper effects [2]. Therefore, novel and efficient analysis techniques are needed for processing and analyzing of high-spatial-resolution remote sensing images. Many studies have been done on segmentation and classification of high-spatial-resolution remote sensing images [2–8]. Tarabalka et al. [4] presented a new spectral-spatial classification scheme for hyperspectral images, combining the pixel-based support vector machine classification results and the watershed segmentation regions together. Bruzzone and Carlin [6] proposed a novel pixel-based system for the classification of high-spatial-resolution images. The spatial context information of each pixel extracted from multilevel segmentation images was used to obtain more accurate and reliable classification result. Chen et al. [2] introduced a modified object-oriented classification algorithm integrating multicharacteristics of high-spatial-resolution remote sensing image. Ünsalan and Boyer [7] put forward a new classification method of land development in high resolution panchromatic satellite images using straight line statistics. Salehi et al. [8] developed a hierarchical rule-based object-based classification framework coupled with height points for complex urban environment classification. The rule set was extracted from a training set of QuickBird image coupled with a layer of height points. In addition, the morphological based segmentation and classification was investigated in the work of Dalla Mura et al. [5] and Pesaresi and Benediktsson [9]. Although some improved algorithms can increase the classification accuracy by making use of spectral and textural information, the classification results still cannot satisfy all actual needs. Most of these methods were developed on fusion images or multispectral images. The spatial relationship of high-spatial-resolution remote sensing image between panchromatic and multispectral bands has not been fully considered.

This paper proposes a new high-spatial-resolution remote sensing image classification method based on a mechanism of spatial mapping and a strategy of reclassification. The analysis makes use of both spectral and spatial information from high-spatial-resolution remote sensing data. The classification framework uses a spatial mapping mechanism to fit the special data format and the content of high-spatial-resolution remote sensing images. This algorithm is not based on a fusion image, but rather on raw high-spatial-resolution remote sensing data, which makes full use of the spatial resolution relationship of panchromatic and multispectral images.

QuickBird and SPOT-5 satellite data were employed in a series of experiments and comparative analyses. Traditional pixel-based SVM, object-oriented SVM, and the method proposed in [4] (SVM + Majority Voting, SVMMV) were used for comparison to analyze the advantage of the proposed method. Experimental results show that a classification method based on a spatial mapping mechanism and reclassification strategy for high-spatial-resolution remote sensing data can make full use of the information in both panchromatic and multispectral bands, integrate the pixel- and object-based classification methods, and improve the classification accuracy.

## 2. Methodology

**Figure 1:**
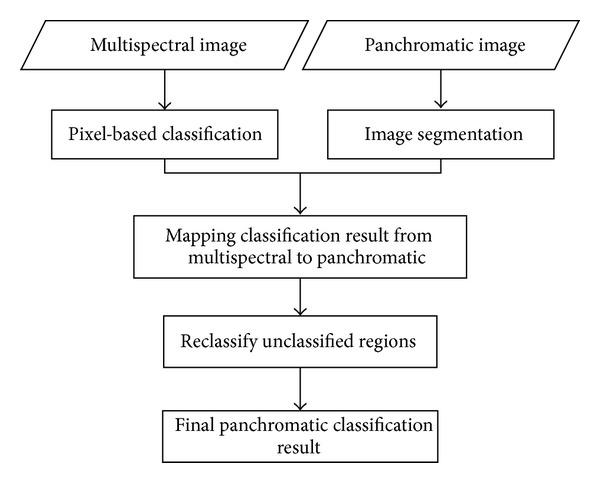
The flow chart of the proposed classification method.

This section details the support vector machine classification method, and watershed image segmentation, and outlines the spatial mapping mechanism and reclassification strategy. A flow chart of the proposed classification method is shown in Figure 1.

### 2.1. Support Vector Machine Classification

The first step in the proposed method is the pixel-based classification of a multispectral image. There are many possible image classification algorithms for remote sensing images. Each algorithm has its unique advantages and weaknesses. In this paper, we focus on the application of the spatial mapping mechanism and reclassification strategy for high-spatial-resolution remote sensing images. In order to improve the overall efficiency of approach and to simplify it, a support vector machine classifier is chosen. Theoretically, any pixel-based classification method can be applied in the proposed method.

Support vector machine (SVM) is a supervised non-parametric statistical learning technique and widely used in classification of remote sensing images [10]. SVM is well at solving nonlinear, high dimensional, and limited training samples [8]. In this paper, LIBSVM library is used to implement the SVM classification algorithm [11].

### 2.2. Watershed Image Segmentation

Image segmentation is an important part of image interpretation, especially for high-spatial-resolution remote sensing images [12, 13]. High-spatial-resolution remote sensing images contain more information of ground objects and show great diversity of them. The purpose of segmentation is to divide an image into homogeneous regions. Watershed transformation is a powerful mathematical morphology technique for image segmentation [4, 14]. A watershed algorithm is a good choice for high-spatial-resolution remote sensing images because of its fast segmentation speed [15].

In this paper, a labeled watershed segmentation algorithm was used to segment the panchromatic image. Morphological operators were used to characterize the gradient of the image, which enabled the watershed segmentation algorithm to label areas within the image.

### 2.3. Spatial Mapping Mechanism

High-spatial-resolution remote sensing data often contain two types of images, those with a single panchromatic band and those with four multispectral bands [16]. For example, QuickBird images have a panchromatic band with a resolution of 0.6 m and four multispectral bands with a resolution of 2.4 m. A panchromatic image of high-spatial-resolution remote sensing data contains most of the spatial information, whereas a multispectral image has most of the spectral information.

In order to make full use of panchromatic and multispectral image information, image fusion is widely used to integrate the two. The fusion algorithm merges the high-resolution panchromatic and low-resolution multispectral imagery to create an enhanced high-resolution multispectral image. Then, the fused image is used in subsequent applications. Note that the fused image is an estimation which may cause spectral distortion and affect the accuracy of classification results [16]. The effect of fusion directly determines the subsequent application accuracy.

**Figure 2:**
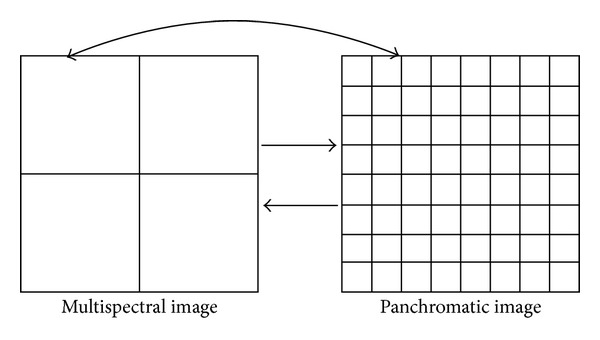
The spatial mapping relationship between multispectral and panchromatic images, taking a resolution rate of 1 : 4 for example.

The proposed classification framework uses a spatial mapping mechanism to make full use of spatial and spectral information in high-spatial-resolution remote sensing data. In the presented method, the raw high-spatial-resolution data, instead of the fusion image, was directly classified based on a spatial mapping mechanism and reclassification strategy. For example, Figure 2 shows the spatial mapping relationship between panchromatic and multispectral images, taking a resolution ratio of 1 : 4. One pixel in the multispectral image corresponds to sixteen pixels in the panchromatic image. For example, if a pixel has position (*x*_*i*_, *y*_*j*_) in the panchromatic image, then *i* = 1,2,…, *m*; *j* = 1,2,…, *n*; the corresponding position of the pixel in the multispectral image is (*x*_*i*_′, *y*_*j*_′), where *x*_*i*_′ = Int(*x*_*i*_/4 + 0.4) and *y*_*j*_′ = Int(*y*_*j*_/4 + 0.4). The Int( ) function rounds a number to the nearest integer.

**Figure 3:**
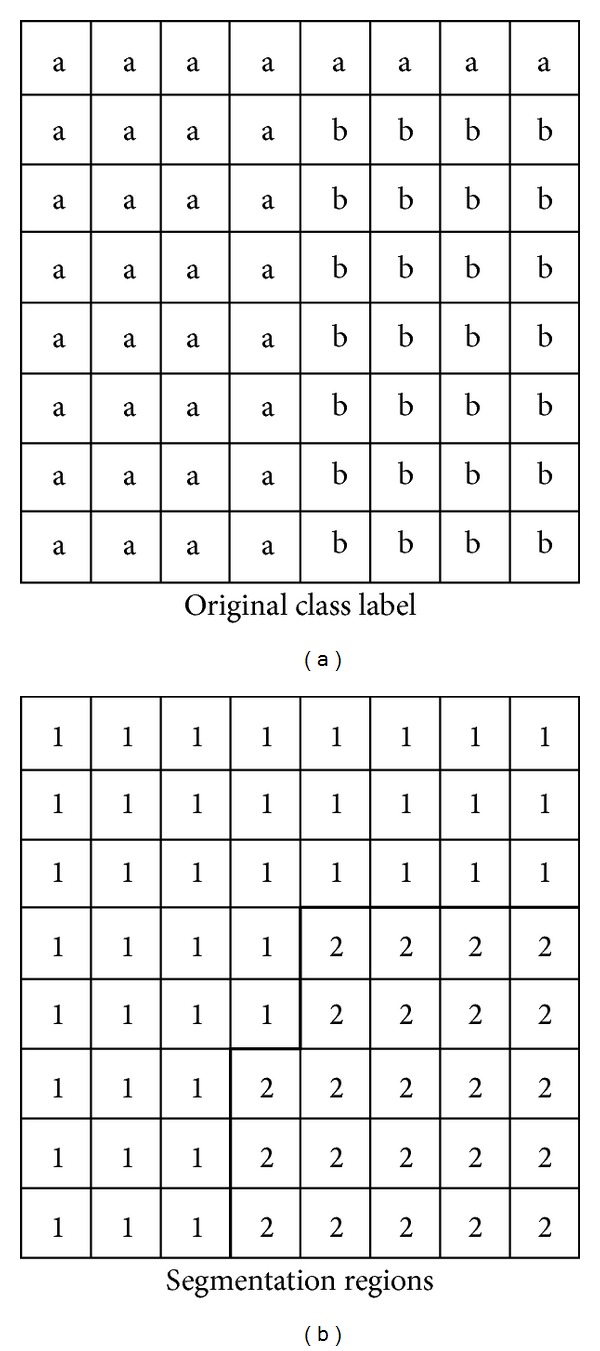
Mapping mechanism by the area dominant principle.

The pixel-based multispectral classification result was mapped to the panchromatic segmentation result based on the spatial mapping mechanism and “area dominant” principle. When using the area dominant principle as a mapping ruler, the areal proportion of each class in each region is computed and a class label corresponding to the maximum area proportion is assigned to that region.  Figure 3 shows the spatial mapping mechanism based on the area dominant principle. On the left is the panchromatic image with the original class labels, whereas spatial mapping is based on the multispectral image pixel-based classification result. On the right are the regions of the panchromatic image segmentation. After spatial mapping based on the area dominant principle, region one is labeled “a” and region two is labeled “b”.

The area proportion of each class represents the region's membership to every class. Regions composed of only one class of pixels have a higher area proportion close to one for this class and zero for other classes. However, regions composed of pixels belonging to several different classes have a lower area proportion for every class. The maximum area proportion of each region reflects the ambiguousness mapping from the pixel-based classification. During the mapping process, an area proportion threshold (0 < *T* ≤ 1) is set. The region is labeled as unclassified if the maximum area proportion does not surpass the threshold. The greater the threshold, the greater the classified regions reliability. In this paper, the threshold was set to 0.6. The regional property is considered very reliable if the maximum area proportion is greater than 0.6. Unclassified regions are reclassified in the next step.

### 2.4. Reclassification Strategy

Unclassified regions in the images will be reclassified based on spectral information using the minimum distance to mean (MDTM) algorithm [17]. The minimum distance classification uses a mean vector for each class and calculates the Euclidean distance from each unclassified region to the mean vector for each class. All unclassified regions were classified to the closest class. In this paper, the classified regions through spatial mapping mechanism based on the area dominant principle were used as training samples.

The regional spectral vector is calculated by the mean spectral vector of pixels contained in each region. The spectral vector of each pixel in a panchromatic image is obtained from a multispectral image by a spatial mapping mechanism.

A MDTM classifier computes the Euclidian distance in spectral space between the mean of every class in the training set and the region to be classified. The Euclidian distance between the mean of a class and an unclassified region in the *n* dimensional spectral feature space is given as [18]

(1) $$\begin{array}{c} D_{\text{ED}}={\left(\sum_{i=1}^{n}{\left(x_{i}-C_{i}\right)}^{2}\right)}^{1/2}, \end{array}$$

where *n* is the dimensionality of data, *x*_*i*_ is the mean spectral value of the *i*th band of the unclassified region, and *C*_*i*_ is the mean spectral value of the *i*th band of one class. The unclassified region is then assigned to the class where *D*_ED_ is minimal.

## 3. Experimental Results and Analysis 

To evaluate the performance of the proposed classification approach, two subsets of high-spatial-resolution remote sensing images, QuickBird and SPOT-5 satellite images, were used in a series of experiments and comparative analyses.

To analyze the advantages of the proposed method for high-spatial-resolution remote sensing images, traditional pixel-based SVM, object-oriented SVM, and the method proposed in [4] (SVM + Majority Voting, SVMMV) on fusion images were used for comparison. The panchromatic and multispectral images were fused by the PANSHARP method in PCI software. Labeled watershed transformation was applied to the morphology gradient of panchromatic image to obtain segmentation regions. The object-oriented SVM was applied on the panchromatic segmentation regions and the region feature was computed from the fusion images. The SVMMV method presented a spectral-spatial classification scheme, combining the pixel-based support vector machine classification results and the watershed segmentation regions through majority voting.

The SVM classifier with Gaussian radial basis function (RBF) kernel was applied in all experiments. The optimal parameters C (parameter that controls the amount of penalty during the SVM optimization) and *γ* (parameter that describes the spread of the RBF kernel) were chosen by fivefold cross validation [11, 19].  Table 1 reports the optimal parameters of all SVM classification experiments.

To assess classification accuracy, a “confusion matrix” is used. Confusion matrices are obtained by selecting points with stratified random sampling and assessing the class of each point as calculated by each of the four methods. The reference classification images were generated through a precise manual interpretation on fusion images. The producer, user, and overall classification accuracies are calculated from the “confusion matrix.”

**Figure 4:**
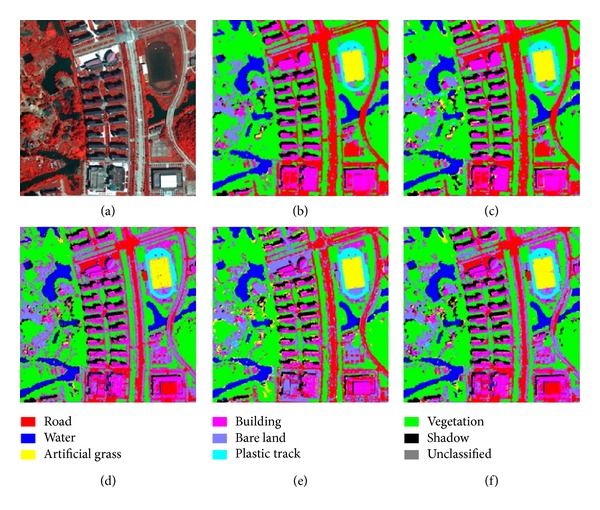
QuickBird images and classification results. (a) is the fused pseudocolor synthetic image, (b) is the primitive mapped classification image, (c) shows the final classification result obtained by the proposed method, and (d) is result of the pixel-based SVM. The classification result of the object-oriented SVM is shown in (e), and the SVMMV is shown in (f).

The first classification experiment was performed with the QuickBird image, including the panchromatic band with a resolution of 0.6 m and four multispectral bands with a resolution of 2.4 m. The size of multispectral image is 256 × 256, whereas that of the panchromatic image is 1024 × 1024. The optimal parameters of SVM classifiers on QuickBird dataset are shown in Table 1. The classification results are shown in Figure 4.  Table 2 shows the producer, user, and overall classification accuracies for the QuickBird Image classification.

**Figure 5:**
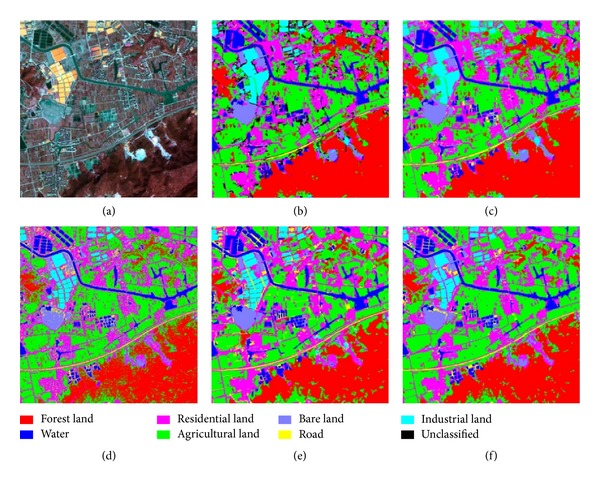
SPOT-5 images and classification results. (a) is the fused pseudocolor synthetic image, (b) is the primitive mapped classification image, (c) shows the final classification result obtained by the proposed method, and (d) is result of the pixel-based SVM. The classification result of the object-oriented SVM is shown in (e), and the SVMMV is shown in (f).

A second classification experiment was performed with the SPOT-5 satellite image, consisting of a panchromatic band with a resolution of 2.5 m and four multispectral bands with a resolution of 10.0 m. The size of the multispectral image is 300 × 300, whereas it is 1200 × 1200 for the panchromatic image. The optimal parameters of SVM classifiers on SPOT-5 dataset are shown in Table 1. The results of classification are shown in Figure 5.  Table 3 shows the producer, user, and overall classification accuracies for the SPOT-5 image classification.

By comparing the accuracies of the various classifications, the proposed method based on a spatial mapping mechanism and a strategy of reclassification can be seen to obtain better classification results than pixel-based SVM, object-oriented SVM, and SVMMV. Tables 2 and 3 show that the overall accuracies of the proposed method are higher than those of the pixel-based SVM, object-oriented SVM, and SVMMV methods. The accuracies of the pixel-based SVM results were lower than other three methods, because the technique suffers from salt-and-pepper effects. Some of the noise in the pixel-based classification result can be reduced through postprocessing (e.g., majority filtering), but postprocessing can result in the dislocation of class boundaries and influence the outcome of subsequent applications. The SVMMV method, which combined the pixel-based SVM classification results and the watershed segmentation regions through majority voting, got higher classification accuracies than pixel-based SVM classification. The salt-and-pepper effects had been reduced and more homogeneous regions were obtained in the SVMMV classification maps. The proposed method obtained higher accuracies than the SVMMV method, because it made full use of the spatial relationship between panchromatic and multispectral images and a strategy of reclassification. Although the object-oriented SVM, pixel-based SVM, and SVMMV classification results were obtained from a very-high-spatial-resolution fusion image, the image was nevertheless an estimation that could introduce spectral distortion and confusion.

**Figure 6:**
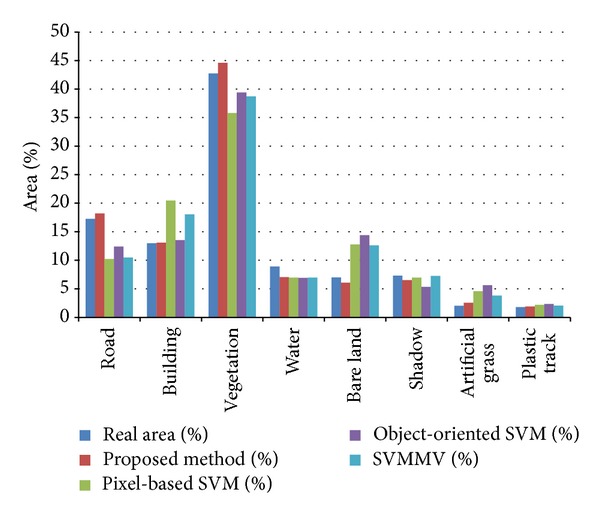
Area statistics of QuickBird data classification results.

In order to make further comparative analysis of the classification accuracy, the area percents of each class for the four methods on the QuickBird dataset were computed (Figure 6). Note that the class area percents of the proposed method are the closest to the real area percents.

According to the experimental results, the proposed classification method based on a spatial mapping mechanism and reclassification strategy can obtain higher accuracy than the pixel-based SVM, object-oriented SVM, and SVMMV classification methods. The proposed method can make full use of the information both in panchromatic and multispectral bands and integrate the pixel-based and object-based classification methods.

In this paper, only spectral features in the images were applied to the classification process. Further work is required to integrate textural features.

## 4. Conclusions 

A new high-spatial-resolution remote sensing image classification method based on a spatial mapping mechanism and reclassification strategy has been presented in this paper, in which a pixel-based classification method and an object-based segmentation and classification method were integrated by a spatial mapping mechanism and reclassification strategy. Furthermore, the proposed method was applied on raw high-spatial-resolution remote sensing data instead of fusion images. Experimental results have demonstrated that the proposed method can make full use of the information in both panchromatic and multispectral bands, integrate the pixel-based and object-based segmentation and classification methods, and obtain higher final classification accuracy.

## Figures and Tables

**Table 1:** Optimal parameters of all SVM classification experiments.

Optimal parameters	QuickBird dataset		SPOT-5 dataset	
	*C*	*γ*	*C*	*γ*
Pixel-based SVM	32768	8	2048	8
Object-oriented SVM	32768	0.03125	2048	0.5
SVMMV	32768	8	2048	8
The proposed method	8192	2	32	8

**Table 2:** Producer and user classification accuracies for QuickBird image classification.

Class	Proposed method		Pixel-based SVM		Object-oriented SVM		SVMMV	
	Producer's accuracy %	User's accuracy %	Producer's accuracy %	User's accuracy %	Producer's accuracy %	User's accuracy %	Producer's accuracy %	User's accuracy %
Road	83.14	80.24	49.72	79.21	55.39	77.88	50.59	79.37
Building	74.93	72.98	64.11	40.80	57.74	55.72	65.96	48.25
Vegetation	88.51	90.07	78.46	93.71	84.48	92.29	83.56	92.56
Water	74.05	96.61	66.97	83.15	74.05	93.33	72.85	91.14
Bare land	57.05	55.71	63.02	35.63	64.93	30.82	64.42	36.03
Shadow	68.00	75.75	63.63	67.66	62.53	83.85	74.33	73.44
Artificial grass	94.78	50.00	91.74	42.71	93.91	34.84	92.61	52.85
Plastic track	91.00	68.16	94.50	77.14	95.50	71.00	96.50	83.19

Average accuracy	78.93	73.69	71.52	65.00	73.57	67.47	75.10	69.60

Overall accuracy	81.01		69.01		72.48		73.04	

**Table 3:** Producer and user classification accuracies for SPOT-5 image classification.

Class	Proposed method		Pixel-based SVM		Object-oriented SVM		SVMMV	
	Producer's accuracy %	User's accuracy %	Producer's accuracy %	User's accuracy %	Producer's accuracy %	User's accuracy %	Producer's accuracy %	User's accuracy %
Forest land	93.00	88.57	77.00	88.51	84.00	83.17	83.00	96.51
Residential land	83.75	87.24	82.14	77.31	92.82	85.79	87.64	82.76
Bare land	64.95	68.48	50.52	64.47	44.33	81.13	46.39	69.23
Industrial land	84.00	93.33	82.00	91.47	89.00	91.75	84.00	95.45
Water	90.00	94.74	88.00	91.67	94.00	87.04	81.00	90.00
Agricultural land	80.33	80.83	80.75	74.14	80.33	81.00	86.13	76.61
Road	69.00	86.72	52.00	69.33	58.00	71.60	54.00	83.02

Average accuracy	80.72	85.70	73.20	79.56	77.50	83.07	74.59	84.80

Overall accuracy	81.32		74.49		77.67		77.69	
